# Integrated care services: lessons learned from the deployment of the NEXES project

**DOI:** 10.5334/ijic.2018

**Published:** 2015-03-30

**Authors:** Carme Hernández, Albert Alonso, Judith Garcia-Aymerich, Anders Grimsmo, Theodore Vontetsianos, Francesc García Cuyàs, Anna Garcia Altes, Ioannis Vogiatzis, Helge Garåsen, Laura Pellise, Leendert Wienhofen, Isaac Cano, Montserrat Meya, Montserrat Moharra, Joan Ignasi Martinez, Juan Escarrabill, Josep Roca

**Affiliations:** Hospital Clínic de Barcelona, Institut d'Investigacions Biomèdiques August Pi i Sunyer, Centro de Investigación Biomédica en red, Enfermedades Respiratorias, University of Barcelona, Master Plan for Respiratory Diseases Ministry of Health Barcelona, Catalonia; Hospital Clínic de Barcelona, Institut d'Investigacions Biomèdiques August Pi i Sunyer, Centro de Investigación Biomédica en red, Enfermedades Respiratorias, University of Barcelona, Barcelona, Catalonia; Centre de Recerca en Epidemiologia Ambiental.Centro de Investigación Biomédica en red de Epidemiologia y Salud Pública, Universitat Pompeu Fabra, Barcelona, Catalonia; Department of Public Health and General Practice, Norwegian University of Science and Technology, Trondheim, Norway; e-Health Unit, Sotiria Hospital, Athens, Greece; Tic Salut. Tecnologia, Innovacio i Salut, Mataró, Catalonia; Agència de Qualitat i Avaluació Sanitàries de Catalunya, Barcelona, Catalonia; 1st Department of Respiratory Medicine, National and Kapodistrian University of Athens, Athens, Greece; Department of Public Health and General Practice, Norwegian University of Science and Technology, Department of Health and Welfare Services, Trondheim, Norway; Centro en Investigación en Economía y Salud-CRES, Universitat Pompeu Fabra, Barcelona, Catalonia; SINTEF, Information and Communication Technologies, Norway; Hospital Clínic de Barcelona, Institut d'Investigacions Biomèdiques August Pi i Sunyer, Centro de Investigación Biomédica en red, Enfermedades Respiratorias, University of Barcelona, Barcelona, Catalonia; Tic Salut, Tecnologia, Innovacio i Salut, Mataró, Catalonia; Agència de Qualitat i Avaluació Sanitàries de Catalunya, Barcelona, Catalonia; Tic Salut. Tecnologia, Innovacio i Salut, Mataró, Catalonia; Hospital Clínic de Barcelona, Institut d'Investigacions Biomèdiques August Pi i Sunyer, Centro de Investigación Biomédica en red, Enfermedades Respiratorias, University of Barcelona, Barcelona; Master Plan for Respiratory Diseases Ministry of Health and Research Network in Chronic Care, Barcelona, Catalonia; Hospital Clínic de Barcelona, Institut d'Investigacions Biomèdiques August Pi i Sunyer, Centro de Investigación Biomédica en red, Enfermedades Respiratorias, University of Barcelona, Barcelona, Catalonia

**Keywords:** case management, integrated health care systems, chronic disease, long-term care, telemedicine

## Abstract

**Objectives:**

To identify barriers to deployment of four articulated Integrated Care Services supported by Information Technologies in three European sites. The four services covered the entire spectrum of severity of illness. The project targeted chronic patients with obstructive pulmonary disease, cardiac failure and/or type II diabetes mellitus.

**Setting:**

One health care sector in Spain (Barcelona) (*n* = 11.382); six municipalities in Norway (Trondheim) (*n* = 450); and one hospital in Greece (Athens) (*n* = 388).

**Method:**

The four services were: (i) Home-based long-term maintenance of rehabilitation effects (*n* = 337); (ii) Enhanced Care for frail patients, *n* = 1340); (iii) Home Hospitalization and Early Discharge (*n* = 2404); and Support for remote diagnosis (forced spirometry testing) in primary care (Support) (*n* = 8139). Both randomized controlled trials and pragmatic study designs were combined. Two technological approaches were compared. The Model for Assessment of Telemedicine applications was adopted.

**Results:**

The project demonstrated: (i) Sustainability of training effects over time in chronic patients with obstructive pulmonary disease (*p* < 0.01); (ii) Enhanced care and fewer hospitalizations in chronic respiratory patients (*p* < 0.05); (iii) Reduced in-hospital days for all types of patients (*p* < 0.001) in Home Hospitalization/Early Discharge; and (iv) Increased quality of testing (*p* < 0.01) for patients with respiratory symptoms in Support, with marked differences among sites.

**Conclusions:**

The four integrated care services showed high potential to enhance health outcomes with cost-containment. Change management, technological approach and legal issues were major factors modulating the success of the deployment. The project generated a business plan to foster service sustainability and health innovation. Deployment strategies require site-specific adaptations.

## Introduction

The Chronic Care model [1] is widely accepted as a conceptual framework to effectively address the burden of Non-Communicable Diseases [2], with Integrated Care Services being one of its core components. However, the practical deployment and extensive adoption of integrated care remain a challenge [3]. Both conceptual traits [4] and practical recommendations [5] of the Chronic Care model were adopted for the design of the current research.

In 2008, the European Union project NEXES [6] was initiated to assess the deployment of four Integrated Care Services supported by Information and Communication Technologies in three sites: Spain (Barcelona), Norway (Trondheim) and Greece (Athens) with different profiles. The underlying common hypothesis was that the transfer of care complexities from hospital-based care to the community using a patient-centred management approach could enhance health outcomes with associated cost-containment. The NEXES project in Barcelona [7] and in Trondheim [8] should be considered as a preliminary initiative of the regional deployment of integrated care.

The four Integrated Care Services evaluated in the project included: Home-based maintenance of rehabilitation effects (wellness and rehabilitation); Enhanced Care for frail patients to prevent hospitalizations; Home Hospitalization and Early Discharge; and Support to remote diagnosis in Primary Care. These four services were chosen because their adequate articulation could cover the longitudinal care requirements of the entire spectrum of severity of chronic patients. Consequently, patients could be at the centre of care.

The original aim of NEXES was to assess the role of technology on the deployment of integrated care; but it evolved towards the evaluation of the effects and barriers for adoption of the four services. It is of note that efficacy of two of the services: Enhanced Care to prevent hospitalizations [9] and Home Hospitalization and Early Discharge [10] had been already demonstrated in two randomized controlled trials conducted in Barcelona well before the project's initiation; whereas the other two services: Home-based maintenance of rehabilitation effects and Support to remote diagnosis were designed for assessment within NEXES. Consequently, evaluation of effectiveness, sustainability and transferability at the European level of these four services were relevant aims in the NEXES project.

The current manuscript describes the deployment process of the four services, as well as their potential for cost-containment. Moreover, it addresses key strategic aspects, namely: service design, organizational aspects, technology, ethical issues and reimbursement modalities, all of which may be useful for a site specific deployment of an IT-supported integrated model of care.

## Material and method

### Service model

We define an Integrated Care Service as a set of well standardized tasks to be provided to a patient on the basis of his/her health condition and social circumstances. The aim is to achieve target objectives aligned with a comprehensive treatment plan. Two differential characteristics of this approach compared to usual care are: (i) its patient centeredness; and (ii) the longitudinal nature of the interventions. The duration of the interventions is dependent on the type of Integrated Care Service provided. One patient can be assigned to one or more integrated care services within a given time frame, depending upon his/her individual needs.

**Figure 4S.**
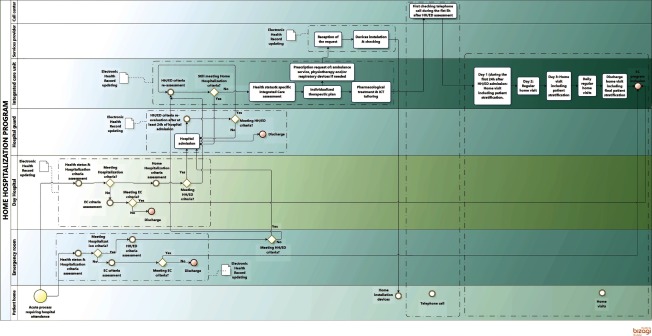
Home Hospitalization and Early Discharge (HH/ED) Patients eligible for the Home Hospitalization/Early Discharge present criteria for hospitalization. The entry into in this service can be through different areas: Emergency room, Day Hospital or general hospitalization ward. The flow of the program is as follows: *i)* the staff of the corresponding department identify eligible patients for Home Hospitalization/Early Discharge and they are offered to be attended at home. If the Home Hospitalization /Early Discharge option is accepted by the patient, the Home Hospitalization/Early Discharge team proceeds to assess and confirm the criteria for admission into the program; *ii)* the patients signs the informed consent; *iii)* the Home Hospitalization/Early Discharge team does a comprehensive evaluation and establish the initial working plan to be developed at home; *iv)* before the transfer at home the patient receives a portfolio including: accessibility to the Call Center; pharmacological treatment that was prepared by the hospital pharmacy; description of the therapeutic plan including a basic educational program; and; logistics for delivery of complementary equipment (sensors, nebulizer, oxygen therapy, ….) depending on treatment plan previously defined; v) an ambulance transfers the patient at home; and, at the end of the process, vi) the patient receive a phone call from the Home Hospitalization/Early Discharge within a period of 5 hours to assess the status of the patient at home and to ensure that the logistics is fully operational. The first home visit by a specialized nurse of the Home Hospitalization/Early Discharge team is carried out within 24 hours followed by a regime of daily visits. The program facilitates respiratory physiotherapy as needed. If the evolution of the patient is not positive, the Home Hospitalization/Early Discharge can program visits to a Day Hospital for assessment or plan a hospital admission. At discharge from Home Hospitalization /Early Discharge, the patients are allocated to the appropriate health care level or included into another Integrated Care Service according to his/her needs and established working plan. A discharge report was prepared by the Home Hospitalization/Early Discharge team. The discharge visit is performed by one Home Hospitalization/Early Discharge team member at patient's home.

**Figure 5S.**
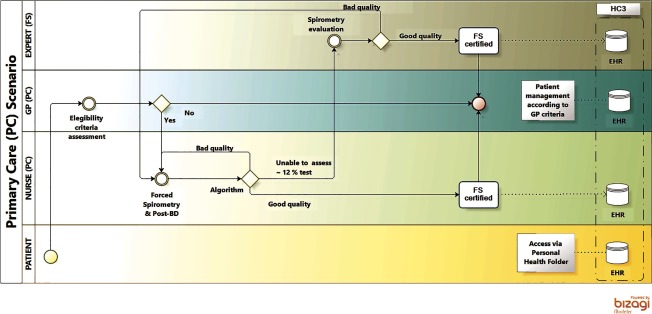
Support to remote diagnosis in Primary Care (Support_1) **Forced Spirometry transfer to Primary Care**. The figure depicts the clinical process of a patient with respiratory symptoms attending a primary care visit. The flow is as follows: the general practitioner decides that the patient is a candidate for Forced Spirometry testing and both baseline and post-bronchodilator studies will be done by a non-specialized nurse. At the end of the testing, the results will be automatically assessed using the algorithm that will generate Forced Spirometry certification for quality. The three possible outcomes are: *i)* the Forced Spirometry testing qualify as high quality. It will be used by the General practitioner for his/her decision-making process and the certified Forced Spirometry will be sent to the patient's Electronic Health Records and to the regional repository (shared Electronic Health Records or “Catalan Electronic Health Records” (Historia Clinica Compartida de Catalunya)); *ii)* the Forced Spirometry testing does not fulfill quality criteria. Then automatic feedback with specific information on the problem is forwarded to the nurse while the patient is still on site. Consequently, the nurse will have the opportunity to solve the problem and generate a high-quality Forced Spirometry test; and, *iii)* approximately 12% of the Forced Spirometry testing will be classified as undefined by the automatic algorithm and forwarded to the specialist for advice. The specialist will provide remote off-line recommendations directly to both the General Practitioner/nurse and the patient will be attended by the General Practitioner who will take the final decision on how to proceed. In two of these scenarios, the first and the third, the certified Forced Spirometry will be forwarded to the regional Electronic Health Records or “Catalan Electronic Health Records” (Historia Clinica Compartida de Catalunya).

**Figure 6S.**
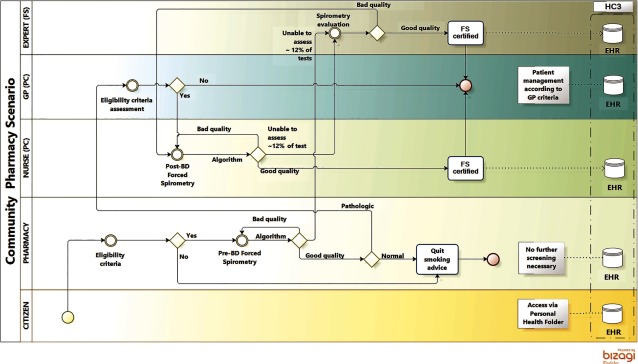
Support to remote diagnosis in Primary Care (Support_2) **Role of Pharmacy Offices (Community Pharmacy) in a Chronic Obstructive Pulmonary Disease case finding program**. The figure depicts the process of a citizen/customer attending a Community Pharmacy where he/she sees a banner inviting participation in a respiratory health status assessment program. If the citizen decides to apply, then the Community Pharmacy officer will administer the Chronic Obstructive Pulmonary Disease questionnaire to assess health status. If risk factors are identified, the citizen will be invited to perform a pre-bronchodilator Forced Spirometry testing carried out by the Community Pharmacy officer. Regarding the quality of the testing, there are three possible outcomes: i) the Forced Spirometry testing is qualified as high quality and it will be certified as such by the automatic algorithm and forwarded to the regionally shared Electronic Health Records *(or* “Catalan Electronic Health Records” (Historia Clinica Compartida de Catalunya); *ii)* the Forced Spirometry testing does not fulfil quality criteria. Then, automatic feedback with specific info on the problem is forwarded to the Community Pharmacy officer while the patient is still on site. Consequently, the Community Pharmacy officer will have the opportunity to solve the problem and generate a high-quality Forced Spirometry test; and, *iii)* approximately 12% of the Forced Spirometry testing will be classified as undefined by the automatic algorithm and forwarded to the specialist for advice. The specialist will provide remote off-line recommendations directly to the Community Pharmacy officer and the certified Forced Spirometry testing will be forwarded simultaneously to the regionally shared Electronic Health Records The citizen's flow in the case of high-quality Forced Spirometry testing can be as follows: *i)* Normal Forced Spirometry testing: the Community Pharmacy officer will generate a report on paper giving tests results and advice about stopping smoking; *ii)* Abnormal Forced Spirometry results: the Community Pharmacy officer will generate a report on paper advising the subject to contact his/her general practitioner, who will have access to the certified Forced Spirometry testing through the “Catalan Electronic Health Records” (Historia Clinica Compartida de Catalunya); and *iii)* Undefined results (12% of the testing): the subject will be informed of the specialist's advice by the Community Pharmacy officer.

Table 1 summarizes outstanding characteristics of the three health systems and the specificities of each site in the project. A high level description of the four Integrated Care Services supported by technology, using a Business Process Management Notation [11], can be found in the online supplementary material, Figures 2S–6S.

### Integrated care services

#### Wellness and rehabilitation

Main objectives of this service [12] were twofold: (i) to achieve long-term sustainability of the training-induced increase of aerobic capacity in clinically stable chronic patients; and (ii) to empower patients for self-management with enhanced daily physical activity and healthier life-styles. All patients were studied at baseline, immediately after an 8-week endurance training programme and at the end of at least 12-month follow-up. After the endurance training programme, the patients were allocated, in a non-randomized manner (Barcelona and Greece), either to intervention (integrated care) or to control (usual care) groups. In Norway, the study was conducted as an individual randomized controlled trial with a 1:1 intervention to control ratio (Table 2). Target variables were aerobic capacity (six minutes walking test) [13], health-related quality of life [14, 15] and daily-life activities (modified Baecke's Questionnaire) [16].

#### Enhanced care for frail chronic patients

In Barcelona, this service was addressed using a three-step approach, as indicated in Table 2. First, we performed a randomized controlled trial assessing effectiveness of prevention of hospitalizations in high risk patients [6]. Thereafter, the site deployed this service as mainstream care in the Integrated Care Unit and later in the Respiratory Department. Finally, we performed a cross-sectional study on patients under Long-term Oxygen Therapy to explore specific functional requirements for the management of frail/complex patients in the community. We identified this niche of patients as representative of the complex transactions among all community stakeholders involved in an integrated care scenario [6]. In Norway and in Greece, this service was assessed using cluster and individual randomized controlled trial designs, respectively.

#### Home hospitalization and early discharge

This service provided an acute, home-based, short-term intervention aiming at fully (Home Hospitalization) or partially (Early Discharge) substituting home care for conventional hospitalization. In Barcelona [6], Home Hospitalization/Early Discharge was deployed as a mainstream service with a real world approach. The service was delivered by trained hospital personnel for a period of time usually not longer than the expected length of hospital stay for the patient's diagnostic-related groups. Target variables in the study were early-readmission rates (30 days) and mortality (Table 2). The Home Hospitalization/ Early Discharge service was assessed as a small randomized controlled trial in Greece. This service could not be deployed in Norway due to organizational factors, as described below.

#### Support for remote diagnosis

The service [17] was conceived as a programme to cut across all areas with the potential to transfer specialized diagnostic capabilities into primary care settings. The studies were initiated using a novel approach providing remote web-based support to primary care settings to achieve high-quality forced spirometry. Eligible subjects were patients with respiratory symptoms who visited Primary Care, and adults at risk for chronic obstructive pulmonary disease, who were offered the test in pharmacy offices (Table 2). The main target variables were achievement of high-quality testing, accessibility to quality-certified testing across the health system and cost savings. In Norway, the service was implemented as a small observational study assessing the potential for performing eco-cardiography with portable equipment in primary care [18].The deployment of the service was not included in the Greek programme because of the characteristics of the primary care setting in the country.

### Characteristics of the three sites

The sites were selected because of three main factors: (i) all of them had a highly motivated leading team willing to explore the complexities of the transition towards integrated care; (ii) represented heterogeneous and characteristic scenarios, as reported below and in Table 1; and (iii) were located in different areas of Europe. The heterogeneity of the sites generated additional complexities during the project development, but it enriched the potential for generalization of the results.

#### Barcelona

The driver of the transfer of complexity was a tertiary public hospital (Hospital Clinic), which had previously set-up a system of coordinated care in one of the four health sectors of the city of Barcelona. NEXES was developed in close alignment with the Health Plan designed by the Department of Health of the Catalan Government [7].

##### Health information sharing platform

Barcelona developed and deployed an open Health Information Sharing platform, Linkcare®, during the project lifetime [19]. Technological delays in the development of wireless mobile technology were the main cause of the pragmatic design of Wellness and Rehabilitation in Barcelona.

##### Organizational setting

Barcelona adopted a building blocks strategy following the principles of the Chronic Care Model [4, 5] and recommendations made by the World Health Organization [4, 5]. Health professionals engaged in the project were those directly involved in the field studies with a leading role of the Integrated Care Unit of the Hospital Clinic. The development of the project was parallel with an extensive workforce reengineering process that was taking place in the health care sector which did not negatively influence the project's organizational setting.

#### Trondheim

The driver for the change in Norway were primary care professionals. NEXES was deployed in the Central Norwegian Region Health Area, at the time of the preparatory phase of Norway's Coordination Reform [8].

##### Health information exchange platform

The ELIN-K® platform was the technological solution used in Norway. It was built on the National framework for messaging and the National secure health net [8]. It is a closed and secure system that connects all health care providers and electronic health record systems. The basic functionality of the ELIN-K® in NEXES was information exchange through electronic messaging across health care providers.

##### Organizational settings

The NEXES team in Trondheim adopted an implementation approach that included simultaneous actions in the different dimensions of the nationwide reform that the Norwegian government was introducing, as described in detail in the “Results” section. In Trondheim, there was an extensive involvement in the project of all the primary care professionals working in the six municipalities that deployed the field studies.

#### Athens

The driver for Greece was a tertiary hospital in Athens, and the IT-supported integrated care services were assessed with small trials related to two main factors: the lack of a fully operational Hospital Information System and the absence of an active governmental plan for deployment of coordinated care nationwide.

##### Technological platform

A simplified version of the Linkcare® platform with no interoperability with corporative electronic health records was used.

##### Organizational settings

Clinical action was taken by a small group of highly qualified and motivated professionals from one large public hospital and one small company devoted to homecare services. The implementation of the services with technological support was done at pilot level.

### Study design and assessment

The initial assessment plan of the NEXES project included randomized controlled trial designs for each of the four services with separate data analysis by site and joint analysis of the three sites for each service. The sample size calculation [20] at site level was obtained considering an intervention to control ratio of 1:1 accepting an alpha risk of 0.05 and a beta risk of 0.20 in a two-sided test and anticipating a drop-out rate of 0.15. However, several barriers that emerged in the very early phases of the project precluded deployment of specific services in Norway and in Greece. Likewise, a pragmatic design for some services was adopted in Barcelona. Deviations from the initial assessment plans, as well as the associated reasons, are described below and summarized in Table 2.

All patients included in the project signed the informed consent after full explanation of the characteristics of the integrated care service administered. Ethical approval was granted by the Ethical Committees from each site. The Model for Assessment of Telemedicine applications [21] was chosen for a systematic analysis and description of outputs for NEXES.

### Statistical analysis

Quantitative analyses of outcomes for each integrated care service were only conducted separately by site. As described above, deviations from the original assessment plan (Table 2) precluded evaluation of each service at project level. Results are expressed as mean ± SD or percentages (%). Distribution of the variables was assessed. Comparison of baseline characteristics between groups was done using parametric or non-parametric tests depending upon the distribution of the variables. Effectiveness of the intervention was tested by comparing outcome variables between intervention and control groups. A cost analysis, direct costs only, of the services was performed and compared to usual care. Analyses were carried out using the statistical package SPSS version 18.0. All analyses were based on bilateral hypotheses with statistical significance set below 0.05.

### Ethical and regulatory issues of technology in the three sites

We focused on the analysis of the European legislation on health data transfer and security, as well as legislative differences by country [22, 23]. The specifics of the sites were analysed with particular emphasis on their potential impact on the deployment of the four IT-supported integrated care services.

## RESULTS

### Assessment of the four integrated care services

The section presents a narrative summary of the overall evidence obtained from the four information technology-supported integrated care services with a global project perspective (Tables 2 and 3). A detailed description of workflows, characteristics of the intervention and outcomes for each service can be found in the online supplementary material.

#### Wellness and rehabilitation

The service demonstrated long-term sustainability of training-induced enhancement of aerobic capacity and had a significant positive impact on life style using simple and robust off-line technological support including the personal health channel as a technological tool to enhance patient adherence to the programme, as described in detail in a previous report [12]. It is of note that the Wellness and Rehabilitation service did not show significant positive effects in two of the sites: Greece and Norway, due to cultural and economic factors not related with the characteristics of the intervention, as discussed below.

#### Enhanced care

The initial randomized controlled trial assessing prevention of hospitalizations in high risk patients [6] showed positive health outcomes in Barcelona and Greece, but not in Norway. Moreover, the deployment of the service as mainstream care in Barcelona generated cost-containments, as indicated in Figure 1S in the online supplementary material. We also identified a high potential for synergies with all NEXES services. The cross-sectional study on patients under Long-term Oxygen Therapy [6] assessed the health status of these patients and identified their health care requirements. The results of the study set the basis for community-based regional deployment of integrated care services for frail chronic patients. Overall, the analysis of the Enhanced Care results recommends the deployment of four different integrated care services under the umbrella of the programme, that is: (i) Prevention of Hospitalization in frail chronic patients with high risk for admissions, as assessed in the project; (ii) Transitional support after hospital discharge; (iii) Community-based integrated care service for clinically stable frail chronic patients; and (iv) Palliative care.

#### Home hospitalization and early discharge

In Barcelona, the deployment of the HH/ED as mainstream service clearly showed that substitution of conventional admissions by home-based hospitalizations should be considered as an option for a high percentage of a wide spectrum of patients that are candidates for tertiary care hospitalization. The service reduced costs both for the health care provider and at the health system level. The small randomized controlled trial carried out in Greece also showed positive outcomes (Table 2). It is of note that Home Hospitalization/Early Discharge was not conducted in Norway because of the obstacles encountered at site level. Briefly, the interplay of three factors: (i) insufficient culture of cooperation between hospital and community-based teams; (ii) existence of two public payers; and (iii) lack of IT tools supporting collaborative work between carers precluded the deployment of the service in Norway.

#### Support to remote diagnosis

The service generated a mature framework for regional deployment aimed at achieving high-quality Forced Spirometry testing in primary care and accessibility to certified forced spirometry testing among service providers across health care tiers [17]. In Norway, this service also showed positive results (Table 2) [18].

### Key results

Overall, the four integrated care services assessed in the project showed high potential to enhance health outcomes with cost-containment. Moreover, safety, acceptability and transferability of the services (Tables 2 and 3) support their potential for large-scale deployment. The following aspects should be highlighted:

- The level of evidence on effectiveness raised in NEXES was uneven for the different services. It is of note that assessment of both wellness and rehabilitation and community-based enhanced care requires further research.
- The observed synergies among different services indicate the need for their implementation in an articulated manner. However, deployment strategies should be adapted to the specificities of the site.
- The standardization of the services’ workflows [19] in the current research facilitates comparability with other deployment experiences

### Lessons learned for the regional deployment

Table 4 displays a decalogue of items that are recommended for regional adoption of integrated care. Findings of our study related to the Model for Assessment of Telemedicine applications dimensions [21] are described below.

#### Technological aspects

The electronic messaging system supported by ELIN-K® fulfilled the legal requirements imposed by the Norwegian legislation, but it showed clear limitations in its ability to support the type of communication among stakeholders across health care tiers required in an integrated care scenario. In contrast, the Health Information Sharing approach [6], Linkcare®, because of its capabilities to embed the service process logic, demonstrated high potential to support the new model of care. Moreover, NEXES provided evidence for the transition from a Health Information Exchange platform to a Health Information Sharing approach required for successful deployment of integrated care [6].

The project also indicated that tele-monitoring should be envisaged as a useful supporting component of a technological approach that must be integrated into the clinical process and modulated by clinical needs. Both patients and professionals showed high degrees of satisfaction with IT functionalities supporting collaborative tools for specific programmes (i.e. mobile videoconferencing and the personal health folder) [6]. The architecture and functionalities of the technological platform developed in Barcelona [19] proved to be a relevant component for the success of the deployment of integrated care, as conceived in the service model depicted in Figure 1.

#### Organizational setting

The deployment strategies undertaken in Trondheim and in Barcelona were markedly different and this had significant consequences for measuring the project outcomes at each site. The NEXES programme in Barcelona was only focused on the assessment of the four integrated care services and it was primarily run through the Integrated Care Unit of the Hospital Clinic. This is a transversal unit supporting specific hospital-based programmes such as Home Hospitalization/Early Discharge and facilitates the bridging between hospital and community care settings, thus fostering the transfer of care complexity from specialized to community care. The setting in Barcelona allowed a focused approach to the specificities of the NEXES aims. In contrast, because of Trondheim's nationwide leadership in Norwegian Health Reform, the team undertook an ambitious programme with simultaneous action in four main areas: (i) deployment of the four services, (ii) implementation of the Health Information Exchange platform; (iii) organizational changes and preparation of the workforce, including dissemination of both clinical and technological models nationwide; and (iv) pioneering the transfer of public hospital care funding from the state to the local authorities for enhanced community care of chronic patients.

#### Ethical and regulatory issues

The Norwegian legislation on data privacy and transfer was identified as a major limitation for the deployment of integrated care in Trondheim, both in terms of the technological approach and design of the clinical interventions. Nevertheless, the project triggered an initiative in the Norwegian Parliament to promote legal changes to facilitate the future deployment of integrated care.

Interestingly, the review of the current European Union legislation on health data sharing did not identify other countries with major limitations for the deployment of integrated care. We observed, however, that sharing of existing standardized data transfer procedures from on-going deployment experiences can favourably contribute to the process of adoption of integrated care.

#### Reimbursement of services and the business model for regional deployment

The four integrated care services assessed in the project showed favourable cost-effectiveness ratios. These positive results were largely due to the avoidance of costly institutional care (hospital admissions) and the transferring of complex services to community providers.

The process of deployment of the integrated care services led to an enrichment of the entire health care value chain with new roles for the existing providers and the emergence of new participants that may generate additional opportunities for team development. The proposed business model (Figure 2) should rely on the relationships of the two core types of stakeholders: (i) the payer(s) and (ii) the health care providers covering different health care tiers. The other components of the value chain (industrial, integrators, operators, etc.) should interact through mainstream health care providers. In systems with two (Norway) or more public payers, strategic agreements favouring a health system vision of the business model are highly advised.

NEXES thoroughly analysed the expected impact of different modalities of reimbursement on the deployment of integrated care services assessing the effects on the business case, their role as incentives for adoption and their potential for generalization at a health system level. Finally, payments by activity and by capitation were discarded, with proposed payments by outcomes using a bundled approach with specific features to ensure service adoption and the take-up of appropriate technological investments (Figure 2). Bundled payment should be perceived as a way to incentivize collaboration among providers in order to move to less intensive and expansive care that would result in better health outcomes. Health care providers would have broader incentives to achieve savings, so that the margins are kept or may even increase. Technological innovation is thus considered part of the bundled payment and not a specific reimbursable charge in the proposed model. The payer would seek an overall reduction in the health care expenditure bill, so that the bundled payment, in the context of a shared-risk scenario, could provide a cost reduction with better quality of care, moving beyond specific interests of any one component of the system.

The business model generated by NEXES was conceived as a recommendation that requires further validation. The aim of the proposal was to facilitate scalability of the deployment of integrated care at health system level. The transfer of approximately 20% of hospital budgetary resources to the community (primary care and convalescence centres) was proposed by the Norwegian Ministry of Health within the Coordination Reform [8]. Likewise, a figure close to 17% is also supported by the literature [24] (Figure 2).

## DISCUSSION

### Relevant findings

The three major achievements of the NEXES project were, first, the demonstration of the effectiveness of IT-supported integrated care services (Tables 2 and 3), with its high potential for cost-containment and complementariness of the deployment of integrated care services assessed in NEXES. The four services should be considered together as a suite of community-based integrated care services covering the spectrum of severity of chronic patients, from citizens at risk through onset of illness to end-stages of disease (Figure 3).

A second achievement was that NEXES demonstrated the relevant role of the technological platform to adapt the services at regional/country levels with strong recommendations for an open source Health Information Sharing approach [19]. Third, the project identified a high degree of transferability of these services and formulated structured strategies adapted to site characteristics that can facilitate regional adoption of integrated care with technological support across Europe.

The project also delineated a realistic and sustainable business model based on bundled payments, with shared-risk fostering investments in integrated care supported by technological innovation with no further increase in the overall health care expenditures.

### Contributions to deployment of integrated care

We acknowledge that the level of maturity of the deployment of the different services was heterogeneous. While *Wellness and Rehabilitation* should be considered in a pilot stage [12], further multicenter validation through a formal cost-effectiveness analysis is needed. In contrast, *Home Hospitalization/ Early Discharge* [6] and *Enhanced Care* for prevention of hospitalizations [6] are already mainstream services at our Hospital Clinic, even though other services under the umbrella of Enhanced Care are still in a development phase [6]. Finally, we must highlight the potential of *Support* Services for remote diagnosis, as it is currently being deployed in the entire Catalan region [17, 25, 26] and almost fully adopted by the Basque Country [27].

We understand that both positive and negative results obtained in the current study have facilitated the identification of modifiable elements and contributed to delineation of site specific deployment strategies aimed at shortening the gap of 7–10 years [28] often seen between initiation of deployment projects and the generation of positive outcomes leading to wide adoption.

The Comparative Effectiveness Research [29] orientation as applied in the project was adapted using the characteristics of the multilevel interventions (clinical, organizational, technological, legal, financial, etc.) in the complex heterogeneous health systems [30]. We believe that the evidence generation process and patient-oriented approach, even with a lack of homogeneous study designs among sites that prohibited joint analyses, support the strength of conclusions obtained with the pragmatic approach chosen for this project.

There are increasing publications generating valuable contributions [31–33] towards the deployment of integrated care services. It is of note that previous deployment experiences, when carried out by a single health care provider, do not generate sufficient evidence for generalization of results to other settings due to the homogeneity of the patients and providers [34–36]. Likewise, the design and results of a recent large randomized control trial, the Whole System Demonstrator [37], conducted in the UK cannot be applied to the entire population. We need expanded information obtained from real world deployment experiences with large heterogeneous groups, like NEXES, to provide evidence for generalization at the European level.

### Regional adoption at European level

The on-going transition towards an integrated care approach in several European Union regions is currently stimulated by three main driving forces. The trigger is, with no doubt, the burden imposed by the epidemics of chronic diseases [2]. But, two additional vectors are accelerating disruptive changes in health care, namely: the need for generating efficiencies allowing further investments for innovation of health care services without increasing overall health costs; and, of equal importance, the paradigm change in understanding the underlying mechanisms of chronic diseases [38–40]. The articulation and site adaptation of the different dimensions analysed in the current research should facilitate the initiatives aiming at regional adoption of this new IT-supported integrated model of care. In the process, however, two factors may likely influence the long-term success of the regional deployment. One of them is the success in implementation of the proposed business model (Figure 2). We acknowledge that the development of a shared-risk approach based on bundled payments may require appropriate interplay between changes in reimbursement policies and research to build-up applicability of the new concepts. Consequently, a transitional phase towards development of the new business models should be envisaged and designed by addressing the dominant barriers at each site. Moreover, the analysis of viability of the business model proposed by NEXES should take into account a recent report indicating that bundle payments applied in a disease-oriented approach resulted in significantly increased costs [41]. A second major element is the need to generate valid tools for long-term assessment of the deployment process (see online supplementary material). In this regard, recent on-going EU initiatives [42] may provide relevant outcomes in this field.

## Conclusions

The research demonstrated that appropriately articulated integrated care services for chronic patients show high potential to enhance health outcomes with cost-containment. Standardization of service workflows facilitates comparability among deployment experiences. Our results identified: technological approach, change management strategy, business plan and legal issues as relevant factors to define site specific strategies for large scale deployment of integrated care. We believe that the project outcomes represent an important contribution towards adoption of integrated care services for chronic patients in Europe.

## Figures and Tables

**Figure 1.**
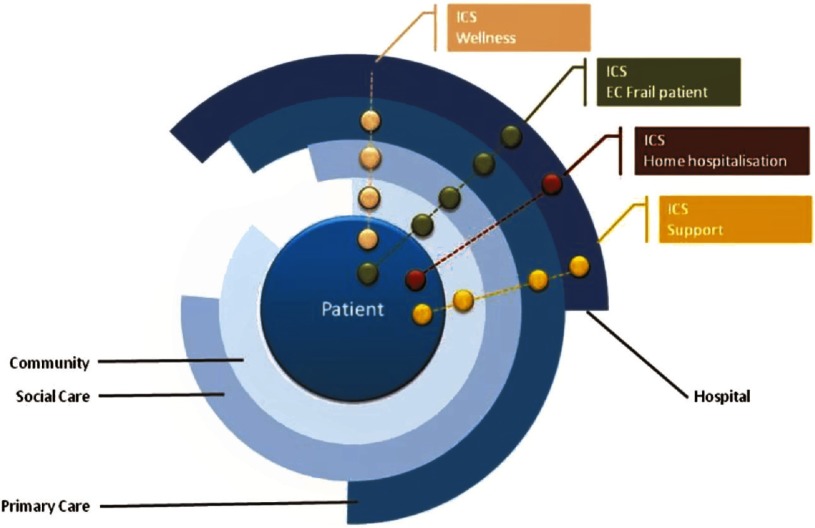
Service model. The family of four Integrated Care Services deployed in NEXES with support of Information and Communication Technologies exemplifies the health paradigm based on longitudinal patient-centred care structured to achieve well-defined objectives with a continuum across the different layers indicated in the figure. Implicit in the model there are shared agreements among actors involving: informal (community) and formal care (primary care and hospital), as well as social support services. Enhanced accessibility of active patients/caregivers and collaborative work among professionals are basic characteristics of the model (see text for further details on the different Integrated Care Services supported by Information and Communication Technologies).

**Figure 2.**
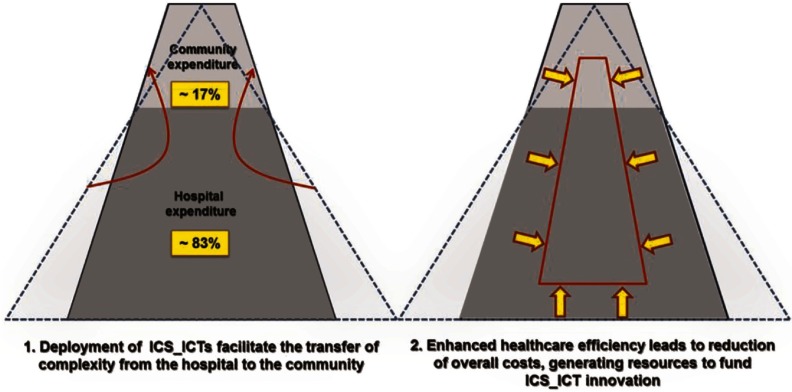
Expected initial effects of the introduction of Bundled Systems with shared risks. The top portion of the left triangle (discontinuous line) indicates the per cent of hospital expenditure (–17%) that can be transferred to the community as Integrated Care Services. Those services are less intensive and less expensive. It will likely enlarge the top portion of the left figure (>17%) narrowing its base (<83%) in order to achieve aggregate cost savings and better margins (for a given reimbursement rate). The right figure displays the expected changes at provider's level after reorganization through Integrated Care Services supported by Information and Communication Technologies. The provider would have broader incentives to achieve savings over time (arrows), so that margins stay larger or increase.

**Figure 3.**
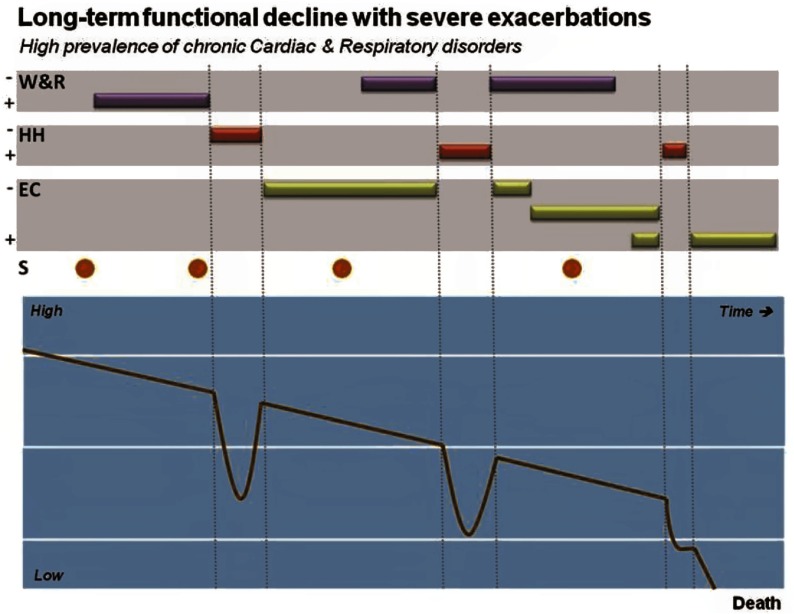
Positioning Integrated Care Services supported by Information and Communication Technologies in chronic patients across time. The four Services were conceived as articulated services covering most of the complexities of chronic patients during the lifetime period. Functional decline overtime and occurrence of exacerbations are common features in chronic patients, acknowledging that both rate of progress and frequency/severity of acute episodes may show large variations among individuals and the characteristics of the predominant disease(s). The different Services can be administered alone or in combination, with different intensities/duration and also different purposes, as displayed. For example, the support to remote diagnosis (S) can be used either for initial diagnosis or for monitoring during the follow-up period.

**Figure 1S.**
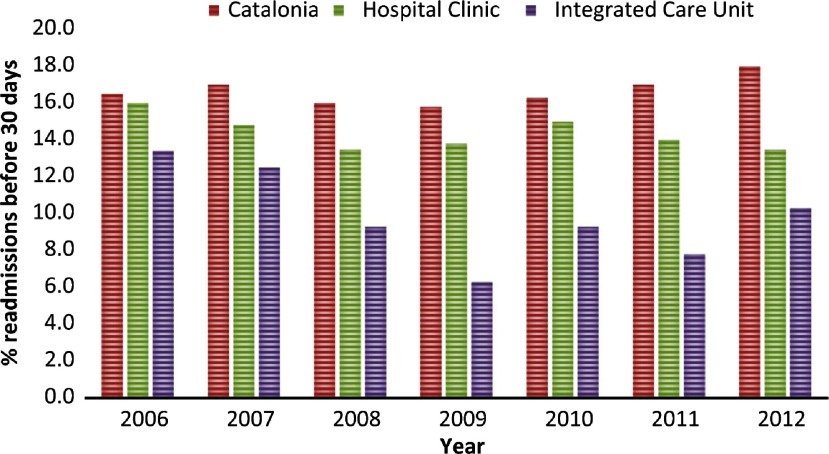
30-day readmissions Comparison of 30-d readmission rates for patients with Chronic Obstructive Pulmonary Disease, expressed as percentages, between the Catalan region, the Hospital Clinic and the Integrated Care Unit at Hospital Clinic *(see text for details)*.

**Figure 2S.**
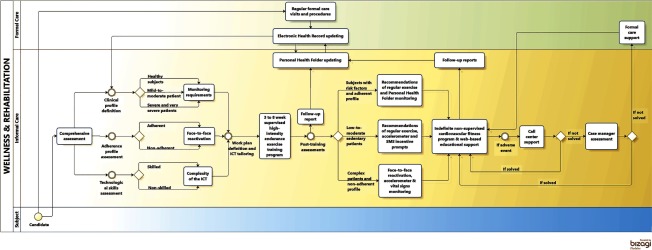
Wellness and Rehabilitation Clinically stable chronic patients (cardiac, respiratory and/or type II diabetes mellitus), at different disease stages, that are eligible for an endurance training program are included into Wellness & Rehabilitation through formal (primary care or specialized care) or informal care (health center, pharmacy offices) providers. Basic assessments at entry into the service are conducted in order to define the work plan based on clinical characteristics, baseline aerobic capacity and adherence profile. Skills and acceptability of supporting technology for the non-supervised period of the program are also evaluated. The patient is then included into a supervised training program (3 to 8 weeks of duration). At the end of the supervised training period, he/she is included into the Integrated Care Service for community-based remotely assisted wellness program managed using his/her personal health folder. Additional supporting technology can be added depending upon requirements and patient's skills. During this non-supervised period, the patient has access to health professionals through the personal health folder and the call center (see text and reference [5] for further details).

**Figure 3S.**
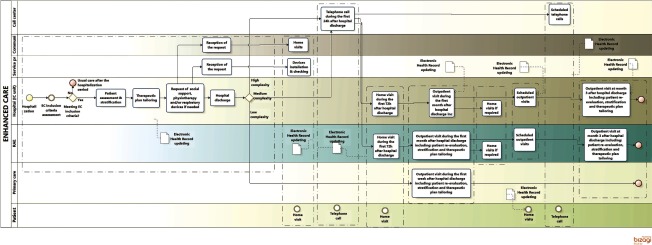
Prevention of admissions in frail chronic patients with previous history of repeated hospitalizations (EC) Patients eligible for the service are frail with previous history of repeated hospitalizations. They can be admitted to the program either from the community or at discharge from an acute event (hospitalization ward, Home Hospitalization/Early Discharge, Day Hospital), as displayed in the figure. The Integrated Care team performs a comprehensive assessment of the eligible patients. During the evaluation process the patient receives a portfolio with: *i)* accessibility to the call center; *ii)* therapeutic plan including pharmacological and non-pharmacological aspects; and, *iii)* provision of supporting technology according to patient's needs established by the therapeutic plan, but also taking into account acceptability and technological skills. During the stud there has been a clear tend to adapt self-monitoring to the real needs of the clinical process as well as to explore tools management tools as the personal health folder. A first visit at home together with primary care professionals was done within 72 hours of admission to the program. No subsequent visits were planned, but the patient had accessibility to program. The latter was customized depending upon the stratification level displayed in the figure.

**Table 1.**
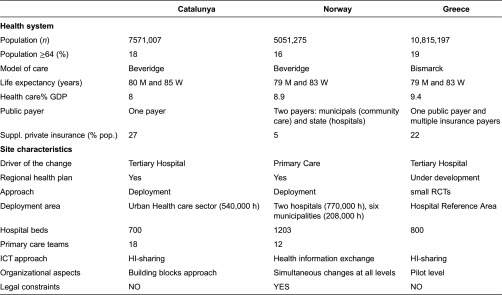
Main characteristics of the sites

M, man; W, women; GDP, gross domestic product; Suppl., supplemental; RCT_S_, randomized controlled trials; ICT, information and communications technology; HI, health information.

**Table 2.**
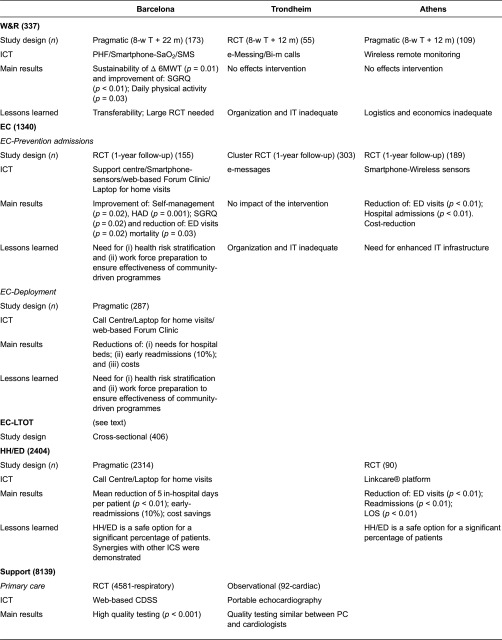

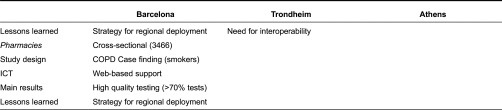
Summary of the field studies assessing the four integrated care services

Number of patients within parenthesis.

COPD, chronic patients with obstructive pulmonary disease; W&R, Wellness and Rehabilitation; EC, Enhanced Care; EC-Prevention Admissions, Prevention of Admissions; EC-LTOT, Long-term Oxygen Therapy; HH/ED, Home hospitalization and Early Discharge; Support, Remote support for diagnosis; 8-w T + Xm, 8-week Training programme and follow-up after training; PHF, Personal Health Folder; ICT, Information and Communication Technology; RCT, Randomized Controlled Trials; SaO_2_, oxygen saturation pulse oximetry; SMS, Short Message Service; e-Messing and Bi-m calls, messaging services using ELIN platform; IT, Information Technology; HAD, Anxiety & Depression; SGRQ, Saint George Respiratory Questionnaire; ED, Emergency Department; web-based Forum Clinic, web-based patient education; CDSS, Clinical Decision Support System.

**Table 3.**
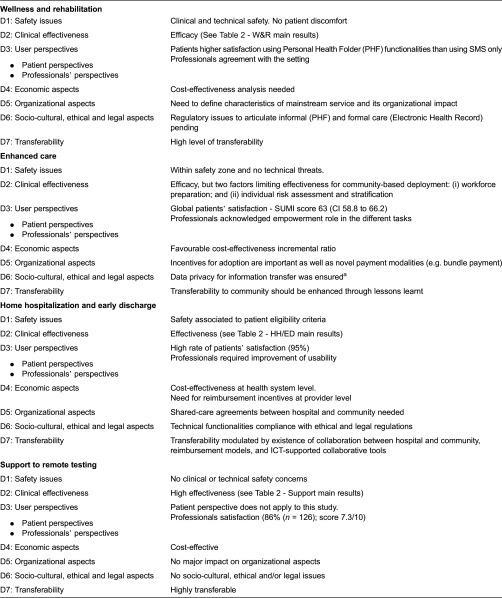
Summary of MAST assessment

^a^Legal frame was a limiting factor in Norway as explained in Lessons Learned.

**Table 4.**
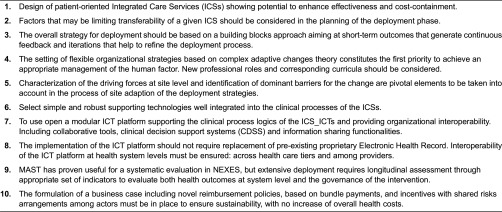
Recommendations for regional deployment of Integrated Care

**Table 1S.**
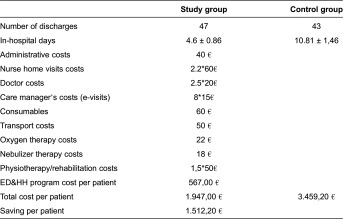
Cost analysis of the HH/ED program in Athens

Early discharge (ED); Home Hospitalization (HH)

## Reviewers

**Georgia L Narsavage**, PhD, APRN, FAAN, FNAP, Director, Interprofessional Education, WVU Health Sciences Center, Professor, WVU School of Nursing, West Virginia University, Morgantown, WV, USA.

**Galileo Pérez-Hernández**, MD, Integrated Medicine specialist, National Center for Preventive Programs and Diseases Control (CENAPRECE). Mexico City, Mexico.

**Francesc Saigi Rubió**, PhD, Telemedicine Programme Director, Department of Health Science, Open University of Catalonia, Catalonia.

**Evi Willemse**, MS, BN, RN, Thomas More University College, Teacher and Researcher, Campus Blairon 800, Turnhout and Ph.D. student, University of Antwerp Campus Drie Eiken Wilrijk, Belgium.

## On-line supplementary material INTEGRATED CARE SERVICES: LESSONS LEARNED FROM THE DEPLOYMENT OF THE NEXES PROJECT

### Introduction

The on-line supplementary material covers 3 main areas, namely: *i)* the first section provides complementary information on outcomes of the four Integrated Care Services supporting the statements formulated in the main manuscript; *ii)* the second section depicts a high level flowchart of each of the four Integrated Care Services supported by Information and Communication Technologies assessed in NEXES using a Business Process Modelling Notation representation; and, *iii)* Finally, we propose basic principles for the assessment strategies to be considered in the regional deployment of integrated care.

Briefly, NEXES was set to explore the practicalities of the Chronic Care model through the assessment of the deployment of four Integrated Care Services supported by technology in three sites with specific profiles. In **Trondheim** (Norway) the transfer of complexity from hospital to home-based services is driven by primary care (municipalities) with a tight involvement in the ongoing Coordination Reform at country level. **Barcelona** (Spain) is representative of a scenario driven by a tertiary care hospital with high potential for scalability at regional level. In **Athens** (Greece), were performed one pragmatic study (Wellness & Rehabilitation) and 2 small randomized control trials: Enhanced Care and Home Hospitalization/Early Discharge in a geographical area wherein formulation of health reform is needed before successful adoption of integrated care can take place. The heterogeneity of the three sites introduced complexities in the take-up of the project, but enriched the potential for generalization of NEXES outcomes at European level.

### Analysis of integrated care outcomes by site

Table 1, in the main document, depicts the basic characteristics of the three sites; whereas Table 2, also in the main document, summarizes the deployment of each of the four services by site. The table indicates: the design of the different studies, number of patients in each of them, type of interventions, technologies used, main outcomes, the impact of each of the studies carried out in NEXES, as well as the references of the results already published. Table 2, also summarizes the main NEXES outcomes in terms of effectiveness and cost-analysis for each the four integrated care services.

In all cases, only cost-analysis of the four services was carried out within the project lifetime. Health outcomes for each of the service were considered in the discussion and final conclusions. We acknowledge that this approach constitutes a limitation of the current study that should be considered as a first attempt toward more complete economic evaluation studies (cost-effectiveness analysis or cost-benefit analysis) in future local and/or regional deployment initiatives in order to compare the costs and consequences of the NEXES services with usual care.

**Wellness and Rehabilitation** – The rationale of the service was that long-term sustainability of training-induced effects on aerobic capacity together with enhanced daily life physical activities are unmet needs in all severity stages of highly prevalent chronic conditions (Chronic Obstructive Pulmonary Disease, Cardiac Insufficiency/Chronic Heart Failure and type II diabetes mellitus). There is evidence that it enhances health outcomes and reduces the use of healthcare resources [1]. Moreover, experimental data seem to support that it might constitute an early preventive strategy modulating disease progress [2]. Previous non-reported data generated by our team [3] indicated that empowerment of patient self-management with technological support may reduce dropouts during the training programs and it may generate long-term sustainability of training effects associated with healthier life style.

In Barcelona, 77 patients with clinically stable Chronic Obstructive Pulmonary Disease finished the 22±12 (19 to 24) months follow-up period after the supervised 8-w endurance training program. The training-induced effects were similar between the intervention and the control groups (p = 0.80). A positive effect of the service at the end of the follow-up period was observed: *i)* preventing decline of aerobic capacity (Δ6Minutes Walking Distance, mean 27 m and -16 m, intervention and controls, respectively, p < 0.01); *ii)* enhancing the activities domain of the Sant George's Respiratory Questionaire [4] (mean -9 and -2, respectively, p < 0.01); and, *iii)* promoting an active life style. The integrated care group reported, at the end of the follow-up, larger amount of hours per week spent on walking activities compared with the control group (2.6±3.8 and 1.3±3.2 h/w) (p = 0.03). We identified the pivotal role of the personal health folder in enhancing patient's adherence and in the achievement of expected outcomes. Wireless remote monitoring during home-based training sessions was needed only in highly selected patients, mainly for safety reasons. Details of the technological platform [5] and complexities of the deployment explaining why only 77 out of 173 eligible patients completed the follow-up were reported elsewhere [6]. As indicated in Table 2, the studies carried out in Trondheim and in Athens did not show significant positive effects of the integrated care services supported by technology due to well identified modifiable factors thoroughly analyzed in [6]. The simplicity of the remote intervention required to support the service suggests that it can be highly cost-efficient, but no formal cost analysis was carried out.

The overall assessment of the service in the 3 sites indicates that the current wellness program should be only taken as a positive feasibility study with enormous potential for chronic care management with a high degree of transferability. Immediate steps should be the design of a large multicenter randomised control trial aiming at consolidating the results obtained in NEXES through a cost-effectiveness analysis, but also assessing additional key aspects to be taken into account for deployment, namely: *i)* criteria for patient stratification of the intervention, *ii)* overall cost and cost components; and, *iii)* confirm practical aspects to be taken into account in a deployment program. A successful outcome in such a large scale randomised control trial would really facilitate a future deployment of this service to be applied in a wide spectrum of subjects with clinical stable conditions, from citizens at risk-patients with mild disease to advanced disease stages.

**Enhanced Care** – Table 2 displays the 3-step strategy followed under the umbrella of Enhanced Care. Firstly, we assessed the potential of generalization of the service addressing prevention of hospitalization in frail chronic patients with previous history of frequent hospital admissions reported in an early randomised control trial done in Barcelona (Catalonia) and Leuven (Belgium) [7] wherein integrated care supported by technology was led by specialized care. To this end, we designed three randomised control trials, one in each site, with specific purposes.

In Barcelona, the study was carried out to analyze effectiveness of the service with a community-based leadership. The intervention strongly reduced both risk of emergency room visits (p = 0.02) and mortality (p = 0.03) and markedly changed the pattern of hospitalizations toward planned admissions, but it did not reduce the total number of hospitalizations. After 12-month of active follow-up, the intervention arm showed a lower percentage of current smokers (control group, 16% *vs* intervention group, 3%, p = 0.02), better knowledge of the disease and self-management in patients with Chronic Obstructive Pulmonary Disease (50% *vs* 71%, p = 0.02), lower depression score (Anxiety and Depression Scale) [8] (mean 7 *vs* 5, p < 0.01) and less symptoms score in the Sant George's Respiratory Questionnaire) [4] (mean 42 *vs* 32, p = 0.02) than usual care. This randomised control trial allowed the identification of two key factors that seem to modulate the success of deployment, that is: adequate patient stratification and preparation of the workforce at community level. A detailed report of the study can be found elsewhere [5].

In Athens, the randomised control trial on prevention of hospitalizations in frail chronic respiratory patients (Enhanced Care n= 92 patients, age 74±4 years and Controls n = 97 patients, age 74±4 years, p = 0.71) generated positive both health and economic outcomes over a 12-m follow-up period. It showed clear beneficial effects of the intervention group on: *i)* Rate of Emergency Room visits (2.7±0.9 *vs*. 5.7±1.3 vs., respectively, p < 0.01); *ii)* Rate of hospital admissions (1.5±0.6 *vs*. 2.7±0.7, respectively, p < 0.01); *iii)* Total in-hospital stay during the year (mean 12 *vs* 31 days, p < 0.001); *iv)* Outpatient visits (8.8±2.0 *vs*11.4±2.6, respectively, p < 0.01); *v)* Health-related quality of life [4] (Sant George's Respiratory Questionnaire, 46±5 *vs*. 52±6, p < 0.01; [9]36-Item Short Form Survey, quality-of-life measures: Physical 55±7 *vs*. 38±13, p < 0.01; and, Mental 50±6 *vs*. 40±12, p < 0.01); and, *vi) C*ost-reduction (mean estimated savings of 3.000 €/year per patient). The lack of effects of this service in Norway (Table 2) may be the end result of a combination of the factors already analyzed in the main manuscript.

The second step of the Enhanced Care strategy undertaken in NEXES had a twofold aims: *i)* the assessment of prevention of hospitalization as mainstream service at Hospital Clinic, and, *ii)* the identification of a suite of services under the umbrella of Enhanced Care aiming at covering unmet needs of chronic patients, as described in the main manuscript. The deployment of the service on prevention of hospitalizations was done in three phases. The initial one (2006) was a small scale deployment in chronic respiratory patients, mostly Chronic Obstructive Pulmonary Disease, within the Integrated Care Unit at Hospital Clinic. This was initiated immediately after the randomised control trial indicated above [7] and in parallel with the extensive deployment of the Home Hospitalization/Early Discharge program covering a wide variety of diagnoses [10]. The strategy was to explore and develop synergies between Enhanced Care and Home Hospitalization/Early Discharge that have shown to be highly positive over the period studied. The combined impact of these two services on early-readmissions is depicted in Figure 1S [11] wherein we compare the evolution of the percentages of 30-day early re-admissions, from 2006 to 2012, among the entire Catalan region, Hospital Clinic and the Integrated Care Unit at Hospital Clinic. At regional level (Figure 1S), the index was stable (mean 16.66%) during the study period, whereas it improved after 2006 at the Hospital Clinic (mean 14.37%, 14%). The Integrated Care Unit showed the rate of early readmissions (mean 9.84%) consistently below the figures displayed by the entire Hospital. In the year 2012, the net rate of early readmissions at the Hospital Clinic was 25% lower than the one seen at regional level. Moreover, the Integrated Care Unit showed a figure 25% below that seen at Hospital level and 43% less than the re-admission rate observed at regional level.

A second phase in the deployment was the remodelling of the hospitalization ward of the Pulmonary Medicine Department by reducing 30% of conventional beds and increasing day hospital activities highly connected with the Integrated Care Unit to facilitate the bridging between hospital-based and community-based care. The change represented a moderate decrease of personnel together with a significant increase of activity mostly due to day hospital visits. Finally, the third phase was undertaken early this year (2014) by extending the program to all types of patients through the organization of transitional care programs encompassing both prevention of hospitalization and transient support post-discharge. As indicated above, the data displayed in Figure 1S represent the combined effects of the two initial phases of the reengineering processes (Integrated Care Unit and Respiratory Department) carried at Hospital Clinic. Likewise, the percentages of readmissions at 90 days for Chronic Obstructive Pulmonary Disease patients were: 23% in Catalonia [11], 35% in Spain [12, 13] and 31% EU level [14, 15].

The third step of the entire Enhanced Care strategy was to explore specific functional requirements for the community-based management of frail/complex patients. To this end, we undertook an observational study (Table 2) including all patients with Long-Term Oxygen Therapy (LTOT) in the Barcelona's healthcare sector assuming that this niche of patients was representative of the complex transactions among all actors involved in an integrated care scenario at community level. A brief description of of the study [10] follows:

The main aims of the study were to assess: *i)* the LTOT program and health status of the patients on LTOT in the health district; *ii)* their frailty profile; and, *iii)* the requirements for effective deployment of integrated care services for these patients. A cross-sectional observational study design including all patients (n = 406) on LTOT living in the health district (540.000 inhabitants). Health status, frailty, arterial blood gases, forced spirometry and hand-grip muscle strength were measured. Network analysis of frailty was carried out. Main study results were: *Adequacy of LTOT prescription:* 47% and 31% of the patients had PaO_2_≤60 mmHg and ≤55 mmHg, respectively. *Adherence to LTOT:* 31% of all patients used LTOT ≥15h/d; this figure increased to 67% in those with PaO_2_≤60 mmHg. *Assessment of frailty:* Overall, LTOT patients presented moderate to severe frailty. *Care complexity* was observed in 42% of the patients. We concluded that adequacy and adherence to LTOT was poor and many patients were frail and complex. The outcomes of the network analysis may contribute to enhance assessment of frailty in LTOT patients. These observations suggest that an integrated care strategy has the potential to improve the health outcomes of these patients.

Overall, the analysis of the three steps of the Enhanced Care results in Barcelona recommend the deployment of four different integrated care services under the umbrella of the program, that is: (i) Prevention of Hospitalization in frail chronic patients with high risk for admissions, as assessed in the project; (ii) Transitional support after hospital discharge; (iii) Community-based integrated care service for clinically stable frail chronic patients; and, (iv) Palliative care. The Enhanced Care program showed effectiveness, high potential for cost-containment as well as for transferability with proper site adaptations.

**Home Hospitalization and Early Discharge** – The deployment of this program in Barcelona as a mainstream service clearly showed that substitution of conventional admissions by home-based hospitalizations should be considered as an option for a high percentage of a wide spectrum of patients that candidate for tertiary care hospitalization. The service generated a mean reduction of 5 days of in-hospital stay per patient resulting in cost-containment both for the service provider and at health system level (Table 2) [10] Briefly, Home Hospitalization and Early Discharge (HH/ED) has proven effectiveness, but its potential as an Integrated Care Service (ICS) supported by Information and Communication Technologies had not been explored. We analyzed effectiveness, costs and barriers for adoption of HH/ED as an ICS into an urban healthcare district in Barcelona.The methodological approach was as follows: (i) Research design: Prospective, pragmatic assessment. Health outcomes from two external control groups were used for post-hoc comparison purposes. (ii) Subjects: Criteria of admission into a tertiary care hospital were required for patient's inclusion. (iii) Measurements: Hospital readmissions, Emergency Department visits and mortality. (iv) Intervention: Home-based individualized care plan, administered as a hospital-based outreach service, aiming at substituting hospitalization and implementing a transitional care strategy for optimal HH/ED discharge. (iv) Statistical analysis: Post-hoc comparisons between HH/ED and each control were done using unpaired Student's t-test or Mann-Whitney test. We included 2,314 admissions (71±15 yrs; Charlson 4±3). In-hospital stay in HH/ED was 1±3 days and the length of home-based stay was 5±3 days. The 30-day readmission rate was 10% and mortality was 3%. Since, the reimbursement rate for HH/ED (918 €/discharge flat rate) was significantly lower than the one paid for conventional hospitalization (2,879€/discharge), HH/ED generated marked cost reduction at health system level, but still a balanced budget at provider's level. The HH/ED demonstrated safety, clinical effectiveness, synergies within the integrated care program, and cost-containment. The program freed an average of 5 in-hospital days per patient. The organizational changes, as well as ethical-regulatory issues, were successfully addressed.

The small randomised control trial on Early Discharge carried out in Athens (Table 1S) (43 and 47 patients with Chronic Obstructive Pulmonary Disease, intervention and controls, respectively) showed excellent clinical results: *i)* lower rate of emergency room visits (0.2±0.4 *vs* 5.7±1.3, p < 0.01), *ii)* lower early re-admissions rate (0.04±0.2 *vs* 2.7±0.7, p < 0.01), *iii)* shorter total length of stay (4.6 ± 0,86 *vs* 10.81 ± 1,46 days, p < 0.01) and, *iv)* cost-savings. The Home Hospitalization/Early Discharge, as defined in NEXES, was not deployed in Trondheim due to two major limiting factors. Firstly, the lack of shared care agreements between hospital and primary care partly explained by the existence of a health systems with two separate payers. The legal constraints in terms of exchange of information sharing across the healthcare system constituted a second limiting factor for deployment of the service in Norway, as analyzed in the main manuscript. Overall, the inclusion of Home Hospitalization/Early Discharge within the family of services to be included in a strategy for extensive deployment of integrated care is highly recommended because it is a safe intervention that generates synergies with other integrated care services and cost-containment at system level.

**Remote support to diagnosis in the community** – The suite of studies [16, 17] under the umbrella the Support service generated the elements required to foster the Catalan deployment of high-quality certified forced spirometry in primary care and pharmacy offices. The cost-analysis carried out in the Basque country [18] represents a low estimate of the potential of the this service for cost-savings. It is of note, however, that the most relevant outcome of the service is its potential for transferability to other diagnostic areas with a marked positive impact on novel diagnostic strategies between specialized and community care. Main conclusions from the deployment of the service (Table 2) in Primary Care and in Pharmacies were:

1. The potential for transferability of Forced Spirometry testing to Primary Care was demonstrated by the effectiveness of the web-based collaborative tool that showed sustainable enhancement of high-quality testing performed by non-specialized professionals.
2. The feasibility of having pharmacy offices play a complementary role to primary care in early diagnosis of chronic respiratory disorders has been proven. Moreover, the potential of pharmacy offices in a future COPD case-finding program has been identified,
3. The lung function testing map in Catalonia was drawn up and requirements for the deployment of high-quality Forced Spirometry within a coordinated care scenario were identified
4. Conventional and novel coaching strategies to be further developed and integrated into the IT-supported platform were analyzed.
5. Technological contributions to health system interoperability aimed at facilitating adoption of a high quality Forced Spirometry service supported by IT were developed and validated, namely: Clinical Document Architecture and an algorithm for automatic assessment of Forced Spirometry quality.

### Business Process Management Notation of the four Integrated Care Services

The standardization of each of the four services includes 6 different sequential blocks explained in detail elsewhere [5], namely: *i)* Case identification, *ii)* Case evaluation, *iii)* Work plan definition, *iv)* Follow-up, *v)* Event handling, and, *vi)* discharge. Case identification refers to the patient's information required at the entry point. Case evaluation includes assessment of the patient to determine his/her eligibility for a given integrated care service and to capture the information required for the next step, work plan definition. The work plan consists on a set of both timed and non-timed tasks, led by a team of healthcare professionals or the patient itself, aligned with the aims of the integrated care for that specific case. Follow-up and event-handling, corresponds to the execution of the working plan. It is of note, that the continuous patient's assessment during this phase may lead to changes in the working plan triggered by unexpected events. Finally, the patient is prepared for his/her discharge from the technological platform. Each of the sequential building blocks of a given typically involves the participation of a limited number of actors. If properly designed, each of these blocks should render outcomes that are relevant for execution of the next phase. Identification of tasks and allocation of them to the different actors was described using the Business Process Modelling Notation [19] formalism. Despite the descriptive part of the Business Process Modelling Notation for each service can be generalized to all settings, the design needs to be contextualized to optimize performance. The Business Process Modelling Notation formalism is used to create the process logic of the technological platform engine and its core functional requirements reported elsewhere [5]. A high level Business Process Modelling Notation description of the 3 Services is depicted in Figures 2S to 6S, namely: Wellness & Rehabilitation^5^ (Figure 2S), Enhanced Care for prevention of admissions for frail chronic patients with high risk of hospitalizations (Figure 3S), Home hospitalization and early discharge (“Hernandez C et al. Lessons from deployment of home hospitalization in a tertiary hospital. Am J Respir Crit Care Med, PA5819 2011”) (Figure 4S) and Support to remote diagnostic in Primary Care [20] (Figure 5S) and Pharmacy Offices [21] (Figure 6S). One of the outcomes of the NEXES project was that Enhanced Care ins an umbrella program that should include four different services, namely: *i)* Long-term prevention of hospitalizations for frail chronic patients with previous history of repeated hospitalizations; *ii)* Short-term transitional care post-discharge; *iii)* Long-term care for frail patients without previous history of hospitalizations; and*, iv)* Palliative care.

### Assessment of the regional deployment of integrated care

The method for assessment of telemedicine applications [22] was useful to provide a systematic description of the evaluation of the four Integrated Care Services during the deployment process. However, as indicated in the main manuscript, NEXES partially infringed one of the basic assessments of telemedicine applications assumptions. That is, the lack of stability of the technological platform during the assessment period. The continuous learning process generated by the project triggered several technological and organizational adjustments with impact on the development of the studies depicted in Table 2.

We learnt that, as soon as positive health outcomes had been demonstrated trough randomised control trials, pragmatic designs were required for evaluation of the mainstream services (Enhanced Care and Home Hospitalization/Early Discharge). Further, a new complex scenario emerges with the assessment of regional deployment of these Integrated Care Services. In NEXES, we have identified two principal challenges to be faced. Firstly, it is clear that randomised control trials allow to generate conclusions with strong internal validity, but they seem to show unacceptable limitations in terms of external validity that make them unpractical for generalization of the results of long-term interventions into to complex adaptive systems. Consequently, there is a clear unmet need to build-up novel assessment tools useful for decision-making after long-term complex interventions into the health systems. Recent initiatives [23] are proposing to generate sophisticated modelling tools based on pre-hoc indicators. The second challenge to be faced is the need to distinguish between the effects that can be attributed to the intervention from other unrelated events that may play as confusing factors. We understand that the problem could be overcome with proper computer modelling techniques taking into account outcome indicators, as well as other factors associated to the specificities of the intervention.

Wellness and Rehabilitation (W&R); Enhance Care for prevention of severe exacerbation (EC-Prev.Hosp); Long term oxygen therapy at home (LTOT); Home hospitalization and Early Discharge (HH/ED); Support to Primary Care (Support to PC); Personal Health Folder (PHF); Information and Communication Technology (ICT); Randomized Controlled Trials (RCT); SaO_2_ (oxygen saturation obtained by pulsioximetry); Short Message Service (SMS); messaging services using ELIN platform (e-Messing and Bi-m calls);
